# Supplementary material for: Vertex protein PduN tunes encapsulated pathway performance by dictating bacterial metabolosome morphology
Source: Nat Commun. 2022 Jun 29;13:3746. doi: 10.1038/s41467-022-31279-3 (PMC9243111; doi:10.1038/s41467-022-31279-3)
Supplement: Supplementary file 1 — Supplementary Information [file 41467_2022_31279_MOESM1_ESM.pdf]

Vertex protein PduN tunes encapsulated pathway performance by dictating bacterial metabolosome morphology

Mills *et al.*

Supplementary Method 1. All-atom molecular dynamics for calculation of bending potential

Calculation of bending potential

The bending potential between the hexamer and pentamer, $V(\theta_B)$, is calculated from the forces on the pentamer at a given bending angle, according to the definition of the force, F_{θ_B}:

$$F_{\theta_B} = -\frac{\partial V}{\partial \theta_B} \quad (1)$$

Here V is the general interaction potential between the hexamer and pentamer, so it is necessary to measure only the forces in the θ_B-direction. Using the GROMACS simulation engine [1], we restrain the position of the PduA hexamer backbone to prevent movement in any Cartesian direction (*x*, *y*, or *z*). In contrast, the PduN pentamer is allowed to move in the *yz*-plane using harmonic restraints. We also restrain the center of mass distance of the pentamer and hexamer in the *z*-direction using a harmonic spring. We run simulations at many different angles, which correspond to different *z*-distances between the centers of mass of the protein. We are thus able to calculate a potential of mean force by performing a discrete summation over those angles

$$V(\theta_{B,n}(z_n)) - V(\theta_{B,0}(z_0)) = -\sum_{i=0}^{n-1} \langle F_{\theta_B}(\langle z_i \rangle)\rangle \left(\langle z_{i+1}\rangle - \langle z_i \rangle\right) \quad (2)$$

$F_{\theta_B}(z)$ is the component of the spring force as logged from the simulations, F_{spring}, in the θ_B-direction:

$$F_{\theta_B}(z) = F_{spring} cos\theta_B \quad (3)$$

Components of F_{spring} perpendicular to the θ_B-direction point directly from the center of the pentamer to pentamer-hexamer interface and are cancelled out by the constraints on the hexamer, F_{hex}(Supplementary Figure 6). Since we can measure the bending angle, θ_B, in the simulation, we can create a one-to-one map between *z* and θ_B, where *z* is the distance between the centers of mass of the pentamer and hexamer (Supplementary Figure 7).

We are careful to use many different "windows" (i.e. make n large and $(z_{i+1} - z_i)$ small) to calculate the mean forces at mean positions in a pseudo-continuous manner with overlap between states, especially near the minimum. In Supplementary Figure 8, each color represents one "window." The same method applies to the case of two hexamers.

The initial configurations for the different windows all use the same starting point—the protein interfaces generated and relaxed as described in the main text. Then, with position restraints on the hexamer (backbone constrained in all Cartesian directions) and pentamer (backbone constrained in *yz*-direction) as described above, but no z center of mass constraint, the *z* center of

mass of the pentamer is pulled at a rate of 1 Å/ns to create configurations with the necessary z values required to generate all the different windows. These simulations take about 20-30 nanoseconds, with independent simulations pulling up and down in the z direction. The pentamer-hexamer interface acts like a hinge and does not detach in this process. Each window is then run for 10 nanoseconds (15 nanoseconds in the hexamer-hexamer case) to compute the mean force, $\langle F_{\theta_B}(\langle z_i \rangle)\rangle$.

A general version of this method is used to calculate the total interaction strength be computing the potential of mean force as a function of the distance, R, between the proteins until they are no longer interacting at R_0.

$$V(R_n) - V(R_0) = -\sum_{i=0}^{n-1} \langle F(\langle R_i \rangle)\rangle \, (R_{i+1} - R_i) \quad (4)$$

Here, $F(R)$ is the force calculated from simulation and does not require adjustments for angular components.

Simulation details

We performed all atom molecular dynamics simulations using the package GROMACS (version 2016.3) [1]. We used the CHARMM36 [2] force field. The recommended CHARMM TIP3P water model [3] was applied with the structures constrained via the SETTLE algorithm [4]. Periodic boundary conditions were employed in all dimensions. The neighbor searching was calculated up to 12 Å using the Verlet particle-based method and was updated every 20-time steps. The Lennard-Jones (LJ) 12-6 interactions were switched off from 10 to 12 Å via the potential-switch method in GROMACS. The short-range Coulomb interactions were truncated at the cut-off distance of 12 Å, and the long-range interactions were calculated using the Smooth Particle Mesh Ewald (PME) algorithm [5], [6]. The NPT ensemble (constant number of particles, pressure, and temperature) was employed. The temperature was coupled using the Nosé-Hover algorithm (characteristic time 1 ps and reference temperature 298K). The isotropic Parrinello-Rahman barostat was employed with the reference pressure of 1 bar, the characteristic time was 4 ps, and the compressibility was 4.5×10^{-5} bar^{-1}. All covalent bonds were constrained, which supported an integration time step of 1 fs. These parameters were recommended for the accurate reproduction of the original CHARMM simulation on lipid membranes [7], and have been verified in simulations on proteins [8], [9], [10], [11] and lipid membranes [12]. All pulling simulations utilize an umbrella pull, where a harmonic potential is applied between the two groups (i.e. pentamer and hexmer). Position restraints also utilize a harmonic potential. The spring constant for these potentials is always 1000 kJ/mol. For the calculation of $\langle F_{\theta_B}(\langle z_i \rangle)\rangle$ over a given window the pull rate is 0 and the harmonic potential restrains the two groups near the initial z value (Supplementary Figure 8).

Calculation specifics

The total interaction of the pentamer-hexamer interface utilizes 24 windows run for 5 nanoseconds each. The bending of the pentamer-hexamer interface utilizes 37 windows. Each window is run for 10 nanoseconds. The bending of the hexamer-hexamer interface utilizes 51 windows run for 15 nanoseconds each.

Differences in number of windows and run time reflect the complexities of the energy landscapes and an effort to reduce error bars relative to the magnitude of the energies. Error bars are based on sampling error and estimated by splitting the data in different sections (first half vs. second half, even data points v. odd data points) and observing the differences in the calculated potential.

Supplementary Method 2. Full mathematical description of systems-level kinetic model

Background

The kinetic, systems-level model for the 1,2-propanediol utilization (Pdu) pathway used in this manuscript was developed based on the original work of Jakobson *et al.* [13], with some modifications. Specific modifications include:

- The assumption that the cytosol of the cell is well-mixed.
- The explicit modeling of the metabolite concentrations in the external media.
- The incorporation of cell growth over time.
- The addition of reaction terms for the conversion of propionyl-CoA into propionate in the cytosol by PduL/PduW.
- The consideration of reverse reaction rates for PduP and PduQ enzymes.

Compared to the previously published work, where analysis focused on the steady-state condition, we focus the analysis in this manuscript on the change in metabolite profiles over time in a batch reactor, as these conditions match those used to generate experimental data.

Model assumptions

We make the following assumptions in our model:

1. At time t, there are $N(t)$ identical, non-interacting cells in a well-mixed solution.
2. The substrates 1,2-propanediol, propionaldehyde, propionyl-CoA, propionate, and 1-propanol passively diffuse across the cell membrane at rates specified by permeability parameters.
3. The substrates 1,2-propanediol, propionaldehyde, propionyl-CoA, propionate, and 1-propanol passively diffuse across the microcompartment (MCP) shell over the entire surface of the spherical MCP at rates specified by permeability parameters.
4. The substrates 1,2-propanediol, propionaldehyde, propionyl-CoA, propionate, and 1-propanol passively diffuse across the microtube (MT) shell along the long axis of the cylindrical MT at rates specified by permeability parameters.
5. There are $n_{\mathrm{MCP/MT}}$ non-interacting MCPs or MTs in the cytosol of each cell.
6. Reactions catalyzed by PduCDE, PduP, and PduQ, forward or reverse, can only occur in the MCP or MT interior.
7. Reactions catalyzed by PduL/W can only occur in the cytosol.
8. The external media, cytosol of the cell, and internal MCP/MT volume are well-mixed such that the concentration in each compartment is assumed uniform at any given point in time.
9. The volume of the external media up to leading order is the volume of the entire culture.
10. The volume of the cytosol up to the leading order is the volume of the cell.

11. All enzymes behave according to Michaelis-Menten kinetics.

Chemical reactions

The following reactions are considered in our model, with the assumptions described above:

$$\text{1,2-propanediol} \xrightarrow{\text{PduCDE}} \text{propionaldehyde}$$

$$\text{propionaldehyde} \xleftrightarrow{\text{PduQ}} \text{1-propanol}$$

$$\text{propionaldehyde} \xleftrightarrow{\text{PduP}} \text{propionyl-CoA}$$

$$\text{propionyl-CoA} \xrightarrow{\text{PduLW}} \text{propionate}$$

Equations used in mathematical model

The differential equations described below were integrated forward in time from a starting condition of 55 mM 1,2-propanediol in the external media, the starting condition for our growth curve.

Concentrations of all substrates are defined by the following variables:

- P_i: 1,2-propanediol concentration in volume i (MCP/MT, cytosol, or external media)
- A_i: propionaldehyde concentration in volume i (MCP/MT, cytosol, or external media)
- Pol_i: 1-propanol concentration in volume i (MCP/MT, cytosol, or external media)
- $PCoA_i$: propionyl-CoA concentration in volume i (MCP/MT, cytosol, or external media)
- $Pate_i$: propionate concentration in volume i (MCP/MT, cytosol, or external media)

Other constants and variables used in the model are defined as follows:

- $V_{\text{compartment}}$: volume of the compartment (MCP or MT)
- $SA_{\text{compartment}}$: surface area of the compartment (MCP or MT); for the MT, this only includes the long axis of the cylinder
- Vol_{cell}: volume of the cell
- SA_{cell}: surface area of the cell
- $Perm_i^j$: permeability of substrate i at interface j (cell surface or MCP/MT surface)
- $R_i(X_j)$: reaction rate if enzyme i as a function of concentrations X in a given volume j (MCP/MT, cytosol, or external media)
- $K_{\text{M}}^{i,j}$: Michaelis constant of enzyme i for substrate j
- $V_{\text{max}}^{i,j}$: Maximum reaction velocity of enzyme i for substrate j
- $N(t)$: Number of cells at time t, calculated from the experimental growth profile
- MT: Refers to MT
- MCP: refers to MCP

- n_{MCP}: Number of MCPs/MTs per cell

The differential equations for the MCP/MT volume are as follows:

$$\frac{dP_{\text{MCP/MT}}}{dt} = -R_{\text{PduCDE}}(X_{\text{MCP/MT}}) + \frac{Perm_{\text{MCP/MT}}^{\text{P}} SA_{\text{MCP/MT}}}{Vol_{\text{MCP/MT}}}(P_{\text{cytosol}} - P_{\text{MCP/MT}}) \tag{5}$$

$$\frac{dA_{\text{MCP/MT}}}{dt} = R_{\text{PduCDE}}(X_{\text{MCP/MT}}) - R_{\text{PduP},f}(X_{\text{MCP/MT}}) - R_{\text{PduQ},f}(X_{\text{MCP/MT}}) + R_{\text{PduP},r}(X_{\text{MCP/MT}}) + R_{\text{PduQ},r}(X_{\text{MCP/MT}}) + \frac{Perm_{\text{MCP/MT}}^{\text{A}} SA_{\text{MCP/MT}}}{Vol_{\text{MCP/MT}}}(A_{\text{cytosol}} - A_{\text{MCP/MT}}) \tag{6}$$

$$\frac{dPol_{\text{MCP/MT}}}{dt} = R_{\text{PduQ},f}(X_{\text{MCP/MT}}) - R_{\text{PduQ},r}(X_{\text{MCP/MT}}) + \frac{Perm_{\text{MCP/MT}}^{\text{Pol}} SA_{\text{MCP/MT}}}{Vol_{\text{MCP/MT}}}(Pol_{\text{cytosol}} - Pol_{\text{MCP/MT}}) \tag{7}$$

$$\frac{dPCoA_{\text{MCP/MT}}}{dt} = R_{\text{PduP},f}(X_{\text{MCP/MT}}) - R_{\text{PduP},r}(X_{\text{MCP/MT}}) + \frac{Perm_{\text{MCP/MT}}^{\text{PCoA}} SA_{\text{MCP/MT}}}{Vol_{\text{MCP/MT}}}(PCoA_{\text{cytosol}} - PCoA_{\text{MCP/MT}}) \tag{8}$$

$$\frac{dPate_{\text{MCP/MT}}}{dt} = \frac{Perm_{\text{MCP/MT}}^{\text{Pate}} SA_{\text{MCP/MT}}}{Vol_{\text{MCP/MT}}}(Pate_{\text{cytosol}} - Pate_{\text{MCP/MT}}) \tag{9}$$

The differential equations for the cytosol of the cell are as follows:

$$\frac{dP_{\text{cytosol}}}{dt} = \frac{n_{\text{MCP/MT}} Perm_{\text{MCP/MT}}^{\text{P}} SA_{\text{MCP/MT}}}{Vol_{\text{cell}}}(P_{\text{MCP/MT}} - P_{\text{cytosol}}) + \frac{Perm_{\text{cell}}^{\text{P}} SA_{\text{cell}}}{Vol_{\text{cell}}}(P_{\text{external}} - P_{\text{cytosol}}) \tag{10}$$

$$\frac{dA_{\text{cytosol}}}{dt} = \frac{n_{\text{MCP/MT}} Perm_{\text{MCP/MT}}^{\text{A}} SA_{\text{MCP/MT}}}{Vol_{\text{cell}}}(A_{\text{MCP/MT}} - A_{\text{cytosol}}) + \frac{Perm_{\text{cell}}^{\text{A}} SA_{\text{cell}}}{Vol_{\text{cell}}}(A_{\text{external}} - A_{\text{cytosol}}) \tag{11}$$

$$\frac{dPol_{\text{cytosol}}}{dt} = \frac{n_{\text{MCP/MT}} Perm_{\text{MCP/MT}}^{\text{Pol}} SA_{\text{MCP/MT}}}{Vol_{\text{cell}}}(Pol_{\text{MCP/MT}} - Pol_{\text{cytosol}}) + \frac{Perm_{\text{cell}}^{\text{Pol}} SA_{\text{cell}}}{Vol_{\text{cell}}}(Pol_{\text{external}} - Pol_{\text{cytosol}}) \tag{12}$$

(13)

$$\frac{dPate_{\text{cytosol}}}{dt} = R_{\text{PduLW}}(X_{\text{cytosol}}) + \frac{n_{\text{MCP/MT}} Perm_{\text{MCP/MT}}^{\text{Pate}} SA_{\text{MCP/MT}}}{Vol_{\text{cell}}} (Pate_{\text{MCP/MT}} - Pate_{\text{cytosol}}) + \frac{Perm_{\text{cell}}^{\text{Pate}} SA_{\text{cell}}}{Vol_{\text{cell}}} (Pate_{\text{external}} - Pate_{\text{cytosol}}) \quad (14)$$

The differential equations for the external media of the cell are as follows:

$$\frac{dP_{\text{external}}}{dt} = N(t) \frac{Perm_{\text{cell}}^{\text{P}} SA_{\text{cell}}}{Vol_{\text{ext}}} (P_{\text{cytosol}} - P_{\text{external}}) \quad (15)$$

$$\frac{dA_{\text{external}}}{dt} = N(t) \frac{Perm_{\text{cell}}^{\text{A}} SA_{\text{cell}}}{Vol_{\text{ext}}} (A_{\text{cytosol}} - A_{\text{external}}) \quad (16)$$

$$\frac{dPol_{\text{external}}}{dt} = N(t) \frac{Perm_{\text{cell}}^{\text{Pol}} SA_{\text{cell}}}{Vol_{\text{ext}}} (Pol_{\text{cytosol}} - Pol_{\text{external}}) \quad (17)$$

$$\frac{dP_{\text{external}}}{dt} = N(t) \frac{Perm_{\text{cell}}^{\text{PCoA}} SA_{\text{cell}}}{Vol_{\text{ext}}} (PCoA_{\text{cytosol}} - PCoA_{\text{external}}) \quad (18)$$

$$\frac{dP_{\text{external}}}{dt} = N(t) \frac{Perm_{\text{cell}}^{\text{Pate}} SA_{\text{cell}}}{Vol_{\text{ext}}} (Pate_{\text{cytosol}} - Pate_{\text{external}}) \quad (19)$$

Reaction rates are assumed to follow Michaelis-Menten kinetics and are defined as follows:

$$R_{\text{PduCDE}} = V_{\text{max}}^{\text{PduCDE}} \frac{P_{\text{MCP/MT}}}{K_{\text{M}}^{\text{PduCDE}} + P_{\text{MCP/MT}}} \quad (20)$$

$$R_{\text{PduP},f} = V_{\text{max}}^{\text{PduP},f} \frac{A_{\text{MCP/MT}}}{K_{\text{M}}^{\text{PduP},f} + A_{\text{MCP/MT}}} \quad (21)$$

$$R_{\text{PduP},r} = V_{\text{max}}^{\text{PduP},r} \frac{PCoA_{\text{MCP/MT}}}{K_{\text{M}}^{\text{PduP},r} + PCoA_{\text{MCP/MT}}} \quad (22)$$

$$R_{\text{PduQ},f} = V_{\text{max}}^{\text{PduQ},f} \frac{A_{\text{MCP/MT}}}{K_{\text{M}}^{\text{PduQ},f} + A_{\text{MCP/MT}}} \quad (23)$$

$$R_{\text{PduQ},r} = V_{\text{max}}^{\text{PduQ},r} \frac{Pol_{\text{MCP/MT}}}{K_{\text{M}}^{\text{PduQ},r} + Pol_{\text{MCP/MT}}} \quad (24)$$

$$R_{\text{PduLW}} = V_{\text{max}}^{\text{PduLW}} \frac{PCoA_{\text{cytosol}}}{K_{\text{M}}^{\text{PduLW}} + PCoA_{\text{cytosol}}} \quad (25)$$

Modified equations for tubes with ends of differing permeability

The differential equations described below were integrated forward in time from a starting condition of 55 mM 1,2-propanediol in the external media, the starting condition for our growth curve. The equations are largely the same as those described above, with some modifications.

The reaction rate definitions remain the same and are thus not repeated here. Notably, there is an additional assumption in this model: That the substrates 1,2-propanediol, propionaldehyde, propionyl-CoA, propionate, and 1-propanol passively diffuse into the MT volume at the ends of the MT at rates specified by permeability parameters.

The variables used in this model are the same as described above, with the addition of two variables—one describing the surface area of the tube ends, and one describing the permeability of the substrates at those tube ends.

- $SA_{\text{MT end}}$: surface area of the ends of a single MT, defined as two times the area of a circle with radius equal to the radius of the MT.
- $SA_{\text{MT axis}}$: surface area along the cylindrical axis of the MT, defined as the circumference of the MT multiplied by the length of the MT.
- $Perm_i^j$: permeability of substrate i at interface j, where now there are three possible surfaces: (1) the cell surface, (2) the surface parallel to the axis of the cylindrical MT (MT axis), and (3) the surface of the MT end (MT end)
- $R_i(X_j)$: reaction rate of enzyme i as a function of concentrations X in a given volume j (MCP/MT, cytosol, or external media) $K_{\text{M}}^{i,j}$: Michaelis constant of enzyme i for substrate j
- $V_{\max}^{i,j}$: Maximum reaction velocity of enzyme i for substrate j
- $N(t)$: Number of cells at time t, calculated from the experimental growth profile
- MT: Refers to MT
- n_{MT}: Number of MTs per cell

The differential equations for the MT volume are as follows:

$$\frac{dP_{\text{MT}}}{dt} = -R_{\text{PduCDE}}(X_{\text{MT}}) + \frac{1}{Vol_{\text{MT}}}\left(Perm_{\text{MT axis}}^{\text{P}} SA_{\text{MT axis}} + P_{\text{MT end}}^{\text{P}} SA_{\text{MT end}}\right)\left(P_{\text{cytosol}} - P_{\text{MT}}\right) \tag{26}$$

$$\frac{dA_{\text{MT}}}{dt} = R_{\text{PduCDE}}(X_{\text{MT}}) - R_{\text{PduP},f}(X_{\text{MT}}) + R_{\text{PduP},r}(X_{\text{MT}}) - R_{\text{PduQ},f}(X_{\text{MT}}) + R_{\text{PduQ},r}(X_{\text{MT}}) + \frac{1}{Vol_{\text{MT}}}\left(Perm_{\text{MT axis}}^{\text{A}} SA_{\text{MT axis}} + P_{\text{MT end}}^{\text{A}} SA_{\text{MT end}}\right)\left(A_{\text{cytosol}} - A_{\text{MT}}\right) \tag{27}$$

$$\frac{dPol_{\text{MT}}}{dt} = R_{\text{PduQ},f}(X_{\text{MT}}) - R_{\text{PduQ},r}(X_{\text{MT}}) + \frac{1}{Vol_{\text{MT}}}\left(Perm_{\text{MT axis}}^{\text{Pol}} SA_{\text{MT axis}} + P_{\text{MT end}}^{\text{Pol}} SA_{\text{MT end}}\right)\left(Pol_{\text{cytosol}} - Pol_{\text{MT}}\right) \tag{28}$$

$$\frac{dPCoA_{\mathrm{MT}}}{dt} = R_{\mathrm{PduP},f}(X_{\mathrm{MT}}) - R_{\mathrm{PduP},r}(X_{\mathrm{MT}}) + \frac{1}{Vol_{\mathrm{MT}}}\left(Perm_{\mathrm{MT\,axis}}^{\mathrm{PCoA}} SA_{\mathrm{MT\,axis}} + P_{\mathrm{MT\,end}}^{\mathrm{PCoA}} SA_{\mathrm{MT\,end}}\right)\left(PCoA_{\mathrm{cytosol}} - PCoA_{\mathrm{MT}}\right) \tag{29}$$

$$\frac{dPate_{\mathrm{MT}}}{dt} = \frac{1}{Vol_{\mathrm{MT}}}\left(Perm_{\mathrm{MT\,axis}}^{\mathrm{Pate}} SA_{\mathrm{MT\,axis}} + P_{\mathrm{MT\,end}}^{\mathrm{Pate}} SA_{\mathrm{MT\,end}}\right)\left(Pate_{\mathrm{cytosol}} - Pate_{\mathrm{MT}}\right) \tag{30}$$

The differential equations for the cytosol of the cell are as follows:

$$\frac{dP_{\mathrm{cytosol}}}{dt} = \frac{n_{\mathrm{MT}}}{Vol_{\mathrm{cell}}}\left(Perm_{\mathrm{MT\,axis}}^{\mathrm{P}} SA_{\mathrm{MT\,axis}} + P_{\mathrm{MT\,end}}^{\mathrm{P}} SA_{\mathrm{MT\,end}}\right)\left(P_{\mathrm{MT}} - P_{\mathrm{cytosol}}\right) + \frac{Perm_{\mathrm{cell}}^{\mathrm{P}} SA_{\mathrm{cell}}}{Vol_{\mathrm{cell}}}\left(P_{\mathrm{external}} - P_{\mathrm{cytosol}}\right) \tag{31}$$

$$\frac{dA_{\mathrm{cytosol}}}{dt} = \frac{n_{\mathrm{MT}}}{Vol_{\mathrm{cell}}}\left(Perm_{\mathrm{MT\,axis}}^{\mathrm{A}} SA_{\mathrm{MT\,axis}} + P_{\mathrm{MT\,end}}^{\mathrm{A}} SA_{\mathrm{MT\,end}}\right)\left(A_{\mathrm{MT}} - A_{\mathrm{cytosol}}\right) + \frac{Perm_{\mathrm{cell}}^{\mathrm{A}} SA_{\mathrm{cell}}}{Vol_{\mathrm{cell}}}\left(A_{\mathrm{external}} - A_{\mathrm{cytosol}}\right) \tag{32}$$

$$\frac{dPol_{\mathrm{cytosol}}}{dt} = \frac{n_{\mathrm{MT}}}{Vol_{\mathrm{cell}}}\left(Perm_{\mathrm{MT\,axis}}^{\mathrm{Pol}} SA_{\mathrm{MT\,axis}} + P_{\mathrm{MT\,end}}^{\mathrm{Pol}} SA_{\mathrm{MT\,end}}\right)\left(Pol_{\mathrm{MT}} - Pol_{\mathrm{cytosol}}\right) + \frac{Perm_{\mathrm{cell}}^{\mathrm{Pol}} SA_{\mathrm{cell}}}{Vol_{\mathrm{cell}}}\left(Pol_{\mathrm{external}} - Pol_{\mathrm{cytosol}}\right) \tag{33}$$

$$\frac{dPCoA_{\mathrm{cytosol}}}{dt} = -R_{\mathrm{PduLW}}\left(X_{\mathrm{cytosol}}\right) + \frac{n_{\mathrm{MT}}}{Vol_{\mathrm{cell}}}\left(Perm_{\mathrm{MT\,axis}}^{\mathrm{PCoA}} SA_{\mathrm{MT\,axis}} + P_{\mathrm{MT\,end}}^{\mathrm{PCoA}} SA_{\mathrm{MT\,end}}\right)\left(PCoA_{\mathrm{MT}} - PCoA_{\mathrm{cytosol}}\right) + \frac{Perm_{\mathrm{cell}}^{\mathrm{PCoA}} SA_{\mathrm{cell}}}{Vol_{\mathrm{cell}}}\left(PCoA_{\mathrm{external}} - PCoA_{\mathrm{cytosol}}\right) \tag{34}$$

$$\frac{dPate_{\mathrm{cytosol}}}{dt} = R_{\mathrm{PduLW}}\left(X_{\mathrm{cytosol}}\right) + \frac{n_{\mathrm{MT}}}{Vol_{\mathrm{cell}}}\left(Perm_{\mathrm{MT\,axis}}^{\mathrm{Pate}} SA_{\mathrm{MT\,axis}} + P_{\mathrm{MT\,end}}^{\mathrm{Pate}} SA_{\mathrm{MT\,end}}\right)\left(Pate_{\mathrm{MT}} - Pate_{\mathrm{cytosol}}\right) + \frac{Perm_{\mathrm{cell}}^{\mathrm{Pate}} SA_{\mathrm{cell}}}{Vol_{\mathrm{cell}}}\left(Pate_{\mathrm{external}} - Pate_{\mathrm{cytosol}}\right) \tag{35}$$

The differential equations for the external media of the cell are as follows:

$$\frac{dP_{\mathrm{external}}}{dt} = N(t)\frac{Perm_{\mathrm{cell}}^{\mathrm{P}} SA_{\mathrm{cell}}}{Vol_{\mathrm{ext}}}\left(P_{\mathrm{cytosol}} - P_{\mathrm{external}}\right) \tag{36}$$

$$\frac{dA_{\mathrm{external}}}{dt} = N(t)\frac{Perm_{\mathrm{cell}}^{\mathrm{A}} SA_{\mathrm{cell}}}{Vol_{\mathrm{ext}}}\left(A_{\mathrm{cytosol}} - A_{\mathrm{external}}\right) \tag{37}$$

$$\frac{dPol_{\mathrm{external}}}{dt} = N(t)\frac{Perm_{\mathrm{cell}}^{\mathrm{Pol}} SA_{\mathrm{cell}}}{Vol_{\mathrm{ext}}}\left(Pol_{\mathrm{cytosol}} - Pol_{\mathrm{external}}\right) \tag{38}$$

$$\frac{dP_{\text{external}}}{dt} = N(t)\frac{Perm_{\text{cell}}^{\text{PCoA}} SA_{\text{cell}}}{Vol_{\text{ext}}}\left(PCoA_{\text{cytosol}} - PCoA_{\text{external}}\right) \quad (39)$$

$$\frac{dP_{\text{external}}}{dt} = N(t)\frac{Perm_{\text{cell}}^{\text{Pate}} SA_{\text{cell}}}{Vol_{\text{ext}}}\left(Pate_{\text{cytosol}} - Pate_{\text{external}}\right) \quad (40)$$

Comparing timescales of diffusion in microtube geometry

The formulation described in the section "Modified equations for tubes with ends of differing permeability" assumes that the interior of the MT is well-mixed, and that, as a result, the driving force for diffusion at the ends of the MT is related to the average concentration in the MT volume. However, given that the length scale along the MT axis is much larger than the radius of the cylinder, we wished to evaluate whether there could be a concentration gradient along this long axis. To this end, we compared the timescale of radial diffusion out of the cylinder to the timescale of diffusion along the long axis of the cylinder, within the MT. As described previously[13], the timescale of diffusion out of an MCP shell can be defined as:

$$\tau_{\text{MCP}} = \frac{R_{\text{MCP}}}{3Perm_i^{\text{MCP}}} \quad (41)$$

Accounting for the change in geometry from spherical to cylindrical, this changes slightly to:

$$\tau_{\text{MT}}^{\text{radial}} = \frac{R_{\text{MT}}}{2Perm_i^{\text{MT axis}}} \quad (42)$$

Assuming a permeability of $10^{-7.4}$ m/s and a MT radius of 50 nm, we find that

$$\tau_{\text{MT}}^{\text{radial}} = 0.6 \text{ seconds} \quad (43)$$

To estimate the timescale of diffusion along the long axis of the MT, we consider the solution of Fick's second law in one dimension, which reads

$$\frac{\partial C}{\partial t} = D\frac{\partial^2 C}{\partial x^2} \quad (44)$$

Where C is concentration of an arbitrary substrate, t is time, and x describes a location in space. We apply the initial condition

$$C(x = 0, t = 0) = M\delta(x) \quad (45)$$

Where M is the total initial quantity of arbitrary substrate added. Using the similarity method, this can be solved to yield the solution

$$C(x,t) = M\frac{1}{\sqrt{4\pi Dt}}\exp\left(\frac{-x^2}{4Dt}\right) \quad (46)$$

To solve for a characteristic time, τ, that it takes, on average, for the mass, M, to travel a given distance, x, we can calculate the expected value $|x|$ as a function of time given this concentration profile over time

$$\langle |x| \rangle = \frac{\int_{-\infty}^{\infty} |x| C(x,t)dx}{\int_{-\infty}^{\infty} C(x,t)dx} \quad (47)$$

$$\langle |x| \rangle = \frac{1}{M}\left(\int_{-\infty}^{0} -\frac{xM}{\sqrt{4\pi Dt}}\exp\left(-\frac{x^2}{4Dt}\right)dx + \int_{0}^{\infty}\frac{xM}{\sqrt{4\pi Dt}}\exp\left(\frac{-x^2}{4Dt}\right)dx\right) \tag{48}$$

$$\langle |x| \rangle = \sqrt{\frac{4Dt}{\pi}} \tag{49}$$

The equation can then be rearranged to calculate the characteristic timescale, $\tau_{\text{diff}}^{\text{axial}}$, it is expected for a concentration spike to disperse to an average length, $\langle |x| \rangle = L$.

$$\tau_{\text{diff}}^{\text{axial}} = \frac{\pi L^2}{4D} \tag{50}$$

Assuming the length scale, L, is the length of a cell (2 µm, in accordance with the data shown in the main manuscript) and D is 10^{-9} m^2/sec[REF CHRIS]

$$\tau_{\text{diff}}^{\text{axial}} = 0.003 \text{ seconds} \tag{51}$$

We note that this is a lower bound on the diffusion time, as the molecules will actually be diffusing in two dimensions rather than just the one considered in this calculation; however, we do not expect the addition of diffusion in the radial direction to increase this time by orders of magnitude. Comparing this, then, to the timescale for diffusion in the radial direction across the MT shell, $\tau_{\text{MT}}^{\text{radial}}$, we find that the timescale of diffusion along the tube axis, as estimated by $\tau_{\text{diff}}^{\text{axial}}$, is two orders of magnitude faster, indicating that the well-mixed assumption should hold, even if substrates must diffuse the entire length of the MT to exit the MT interior.

Supplementary Discussion 1. Systems-level kinetic model

Discrepancies between model and experimental data

In the main text, we noted that there were two main discrepancies between the systems-level kinetic model and our experimental data: (1) propionate and 1-propanol are eventually consumed in our experiments, and (2) absolute propionaldehyde concentrations observed differ from those predicted in the model. To point (1), this discrepancy is expected, as there are no terms in our model that account for the uptake of propionate into central metabolism, which is what occurs in our experiments. Development of the model to accurately capture this phenomenon is certainly of interest for future study, but does not strongly impact the differences we wish to explore here, which are the changes in propionaldehyde buildup in our different compartment geometries. We can be confident that down-stream reactions have low impact on propionaldehyde concentrations because of our sensitivity analysis. The main way in which we expect that excess propionate and 1-propanol would affect propionaldehyde is through the reverse reactions. However, varying the rate of the reverse reaction in the sensitivity analysis has negligible effect on peak propionaldehyde concentration (Supplementary Fig. 4 and 5). So, we expect that including propionate and 1-propanol consumption in the model would have negligible effect on the propionaldehyde peak. To point (2), it has been noted in previous growth studies that the observed propionaldehyde level in the media can be impacted by a suite of growth conditions (temperature, shaking speed), due to the volatility of propionaldehyde [14]. Further, it is also possible that propionaldehyde is being acted upon by other enzymes in the cytosol of the cell, further decreasing the experimentally observed quantity of propionaldehyde in the media. It is thus reasonable to expect that the observed propionaldehyde level in the media may be lower than predicted by our model.

Calculating the number of Pdu MTs assuming either volume or surface area matches that of Pdu MCPs

We noted in the main text that we compared the base MCP case to the MT case by keeping as many parameters the same in the MT model, and that this included total enzyme number per cell. We then considered two limiting cases—one in which total compartment surface area is the same in both MCP and MT models (resulting in an increased enzyme concentration in MTs), and a second in which total volume is the same in both MCP and MT models, which keeps the enzyme concentration the same in both geometries, but results in increased surface area in the MTs. We calculated the number of MTs per cell in these two limiting cases. First, if MCP surface area is conserved (*i.e.*, all shell proteins that make up the shell of 15 MCPs will contribute to MT surface area), then there would be 2.4 Pdu MTs per cell containing 54% of the original MCP volume. Alternatively, if MCP internal volume is conserved (*i.e.*, all enzymes inside 15 MCPs are now inside MTs, and requisite shell protein is assumed available), then there would be 4.4 MTs per cell encased in 1.9 times the original MCP surface area.

Sensitivity analysis of systems-level model

We used a local sensitivity analysis to identify enzyme and compartment features that had the highest impact on propionaldehyde buildup in the two cases (1) spherical MCPs and (2) Pdu MTs with increased surface area (the constant volume case described in the main text). We varied parameters by 10% and observed the resulting maximum propionaldehyde concentration for both models. This analysis revealed that there were two common features in both geometries that determined propionaldehyde buildup outside of the cell: (1) the kinetics of the PduP and PduQ enzymes, and (2) the overall transport of substrates in and out of the compartment volume (Supplementary Fig. 4 and 5). The decrease in propionaldehyde buildup with enhanced PduP/PduQ activity (either by increasing V_{max} or by decreasing K_{M} for the forward reaction) is expected, as PduP and PduQ both have relatively slow kinetics compared to PduCDE, which rapidly generates propionaldehyde (resulting in buildup) [13], [15], [16], [17]. The main compartment features that influence peak propionaldehyde level are compartment surface area and permeability—as the compartment surface area or permeability increases, the access of PduCDE to the 1,2-propandiol substrate increases, resulting in more rapid and thus substantial buildup of the toxic propionaldehyde intermediate. Interestingly, in the Pdu MT constant volume case where there is increased surface area (Supplementary Fig. 5), the peak propionaldehyde level is also sensitive to the V_{max} (forward) of the PduCDE enzyme. This is distinct from the spherical MCP case, where the kinetics of the PduCDE enzyme had little effect on peak propionaldehyde level. This is because the increased surface area that the Pdu MTs have in the constant volume case (1.9 times higher than that of spherical MCPs) increases the overall flux of 1,2-propanediol into the compartment volume, shifting the system into a regime where the rate of the reaction mediated by the PduCDE enzyme is similar to the rate at which 1,2-propanediol enters the compartment. In other words, higher surface area Pdu MTs are no longer rate limiting and the reaction mediated by the PduCDE enzyme becomes rate limiting.

Considering the substrate transport out the ends of Pdu MTs

Based on the various characterizations shown in Figure 2 of the main text, it is unclear what the nature of the ends of Pdu MTs look like with respect to providing a diffusive barrier for substrates. To gain a basic understanding of how varying permeability at tube ends might impact pathway performance, we modified our MT model by adding the tube ends as a surface across which substrates could diffuse. We set the permeability at the tube ends as a separate parameter from the permeability across the MT shell to allow for the possibility that MT ends could be essentially open to the cytosol. Notably, the modified model assumes the MT volume is well-mixed (see "Modified equations for tubes with ends of differing permeability" in Supplementary Method 2), which is appropriate for the time scale associated with diffusion along the length of the tube (see "Comparing timescales of diffusion in microtube geometry" in Supplementary Method 2). Within this framework, changes in permeability at the tube ends only changes the average or effective permeability in and out of the MT.

Given the size of the MTs assumed for our model (50 nm diameter, 2 μm long), including tube ends as a surface for substrate diffusion increases the total MT surface area by 1.2%. If we assume that the MT ends have a permeability equal to that of the rest of the MT, the change in metabolite profiles is minimal (Supplementary Figure 9a), which makes sense given the relatively small overall change in MT surface area. However, if we instead assume that free diffusion can occur at MT ends (corresponding to a permeability of 10 m/s at this surface [18]), a large shift in the metabolite profiles occurs, in which 1,2-propanediol is consumed rapidly, leading to a large buildup of propionaldehyde (Supplementary Figure 9a). While initially surprising, this makes sense when considering that allowing free diffusion at a surface increases the permeability of this surface by ~8 orders of magnitude. Notably, this observation does not agree with our experimental results, which indicate that propionaldehyde buildup is limited by MT structures (Figure 4). To better understand what permeability at MT ends would be consistent with the experimental data presented in the manuscript, we documented the maximum propionaldehyde concentration predicted by the model as a function of the permeability at MT ends (Supplementary Figure 9b). This analysis revealed that there must be a substantial diffusive barrier at the MT ends to suppress propionaldehyde buildup in the external media. Specifically, a permeability less than 10^{-6} m/s is required to keep the predicted maximum propionaldehyde level below 20 mM. Because the *in vivo* structural analysis techniques employed in this study do not permit discernment of the exact structure or arrangement of MT ends, we are do not wish to speculate on what may be serving as a diffusive barrier at MT ends. However, we believe that emerging structural analysis techniques as well as study of non-native pathways in these MTs may yield insights on this front in future studies.

Supplementary Discussion 2. Glycine 52 point mutant to asparagine

We noted in the main text that unlike other PduN point mutants at residue G52, PduN-G52N has a mixed population of MCP and MT-like structures. These results suggest that the PduN-G52N is able to facilitate Pdu MCP vertex capping to some extent. This finding was initially surprising, as the asparagine side chain is more dissimilar to the native glycine residue than a smaller point mutant like alanine. However, a more detailed look at the crystal structure of the pentamer-hexamer interface in the crystal structure of homologous shell proteins reveals that the backbone carbonyl in the G52 equivalent residue participates in a hydrogen bond with a lysine on the hexamer, suggesting that this hydrogen bond formation contributes to the stability of this interface [19], [20]. We thus hypothesize that the amidic carbonyl group in the asparagine side chain contributes to a similar hydrogen bonding interaction [21] that partially compensates for the steric penalty associated with inserting a large side chain in this position.

Supplementary Table 1. Parameters used in systems-level kinetic model for calculations of metabolite profiles over time when the Pdu pathway is encapsulated in MCPs or MTs

**Parameter Name**	**MCP Case**	**MT Case, surface area same as MCP**	**MT Case, volume same as MCP**	**Unit**
CDE_con	0.462	0.863	0.462	mM
CDE_tot [22]	6000	6000	6000	enzymes/cell
cell_length	2.47E-06	2.47E-06	2.47E-06	m
cell_radius	3.75E-07	3.75E-07	3.75E-07	m
cell_surface_area	5.82E-12	5.82E-12	5.82E-12	m^2
cell_volume	9.81E-19	9.81E-19	9.81E-19	m^3
external_volume	5.00E-05	5.00E-05	5.00E-05	m^3
kcatCDE [15]	300	300	300	1/s
kcatL	100	100	100	1/s
kcatPf [16]	55	55	55	1/s
kcatPr [16]	6	6	6	1/s
kcatQf [17]	55	55	55	1/s
kcatQr [17]	6	6	6	1/s
L_con [23]	0.1	0.1	0.1	mM
mcp_surface_area	6.16E-14	3.88E-13	3.88E-13	m^2/tube
mcp_volume	1.44E-21	4.85E-21	4.85E-21	m^3/tube
Navogadro	6.02E+23	6.02E+23	6.02E+23	molecules/mole
Nmcp	15	2.38	4.44	MTs per cell
P_con	0.694	1.295	0.694	mM
P_tot [22]	9000	9000	9000	enzymes/cell
PermMCPNonPolar	3.98E-08	3.98E-08	3.98E-08	m/s
PermMCPPolar	3.98E-08	3.98E-08	3.98E-08	m/s
Q_con	0.520	0.971	0.520	mM
Q_tot [22]	6750	6750	6750	enzymes/cell
radius_mcp [24]	7.00E-08	2.50E-08	2.50E-08	m
VmaxCDEf	138.74	258.98	138.74	mM/s
VmaxLf	10.00	10.00	10.00	mM/s
VmaxPf	38.15	71.22	38.15	mM/s
VmaxPr	4.16	7.77	4.16	mM/s
VmaxQf	28.62	53.41	28.62	mM/s
VmaxQr	3.12	5.83	3.12	mM/s
KmCDEPropanediol [15]	0.5	0.5	0.5	mM
KmPfPropionaldehyde [16]	15	15	15	mM
KmPrPropionyl [16]	95	95	95	mM
KmQfPropionaldehyde [17]	15	15	15	mM
KmQrPropanol [17]	95	95	95	mM
KmLPropionyl	20	20	20	mM
PermCellPropanediol [25] [26] [27]	1.00E-04	1.00E-04	1.00E-04	m/s
PermCellPropionaldehyde [25] [26] [27]	1.00E-02	1.00E-02	1.00E-02	m/s
PermCellPropanol [25] [26] [27]	1.00E-04	1.00E-04	1.00E-04	m/s
PermCellPropionyl [25] [26] [27]	1.00E-05	1.00E-05	1.00E-05	m/s
PermCellPropionate[25] [26] [27]	1.00E-07	1.00E-07	1.00E-07	m/s

Supplementary Table 2. Number of cells counted for each replicate experiment shown in Figure 7b.

	Cells Counted per Replicate			
**Genotype**	**1**	**2**	**3**	**Total**
WT (G52 Panel)	58	81	44	183
ΔPduN (G52 Panel)	28	37	39	104
G52A	47	97	35	179
G52C	19	36	48	103
G52D	30	68	76	174
G52E	49	83	26	158
G52F	111	104	34	249
G52H	22	38	40	100
G52I	43	68	80	191
G52K	54	46	65	165
G52L	46	73	33	152
G52M	42	47	62	151
G52N	54	59	101	214
G52P	42	61	47	150
G52Q	93	43	70	206
G52R	30	108	63	201
G52S	38	66	48	152
G52T	37	53	58	148
G52V	49	50	48	147
G52W	26	55	48	129
G52Y	58	84	59	201
WT (T88 Panel)	63	51	72	186
ΔPduN (T88 Panel)	69	54	45	168
T88A	61	82	71	214
T88C	72	71	85	228
T88D	85	104	84	273
T88E	61	70	70	201
T88F	89	93	72	254
T88G	73	96	75	244
T88H	63	55	66	184
T88I	52	63	65	180
T88K	51	57	100	208
T88L	145	86	195	426
T88M	95	117	83	295
T88N	84	71	56	211
T88P	96	108	57	261
T88Q	78	56	135	269
T88R	98	85	76	259
T88S	95	71	94	260
T88V	113	70	89	272
T88W	78	80	85	243

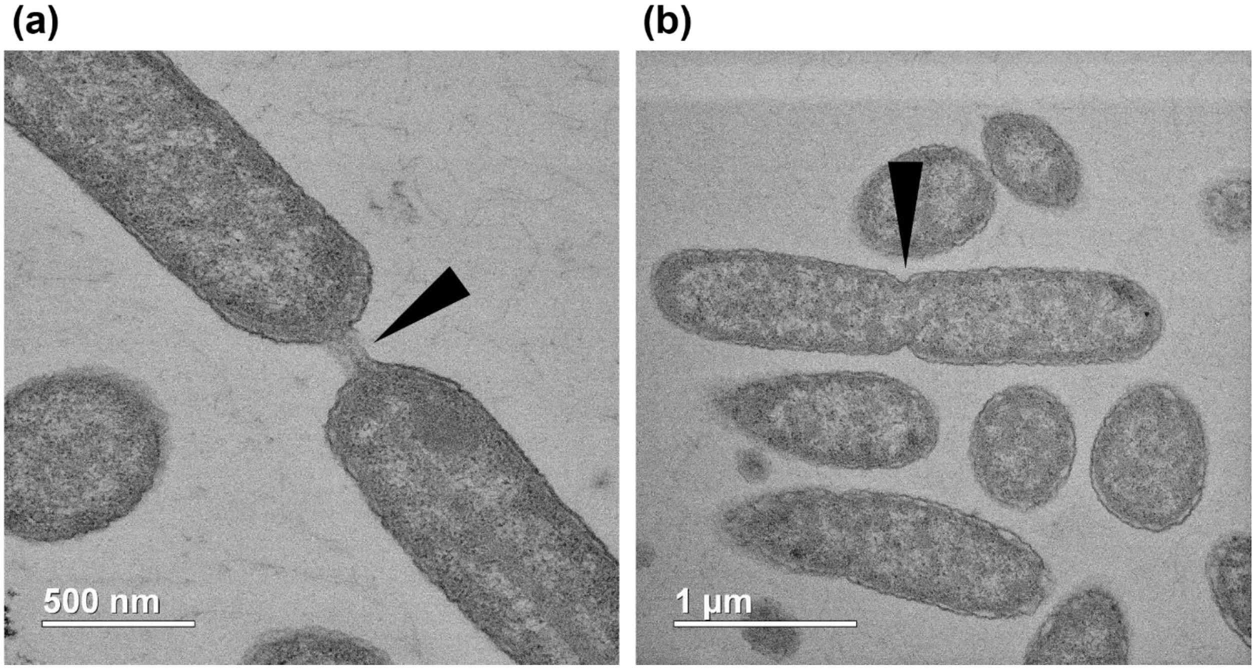

Supplementary Figure 1. Transmission electron micrographs of thin cell sections of *S. enterica* LT2 expressing the *pdu* operon in (a) a PduN knockout strain and (b) a wild type strain. Arrows indicate a cell division event. In the PduN knockout strain (a), Pdu MTs can be seen blocking cell division, whereas Pdu MCPs in the wild type strain (b) do not cause cell division defects. Similar structures were observed in multiple cells in the same sample, but due to cost constraints, this experiment was only performed on one biological replicate.

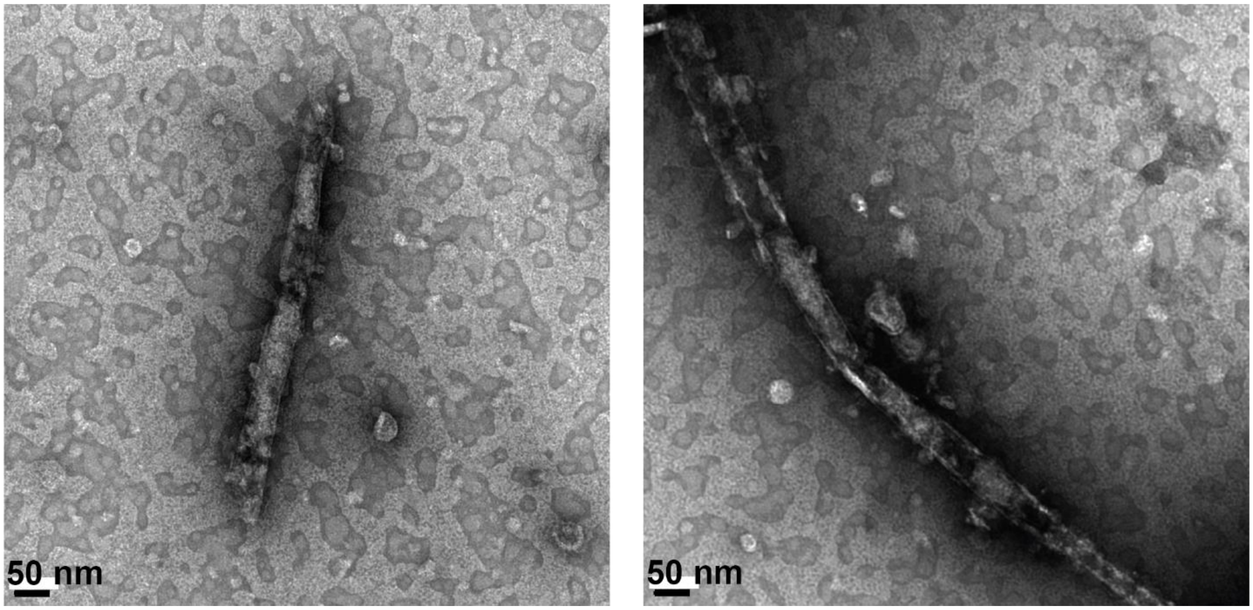

Supplementary Figure 2. Additional transmission electron micrographs on Pdu MTs. Similar structures were observed across experiments performed in biological triplicate.

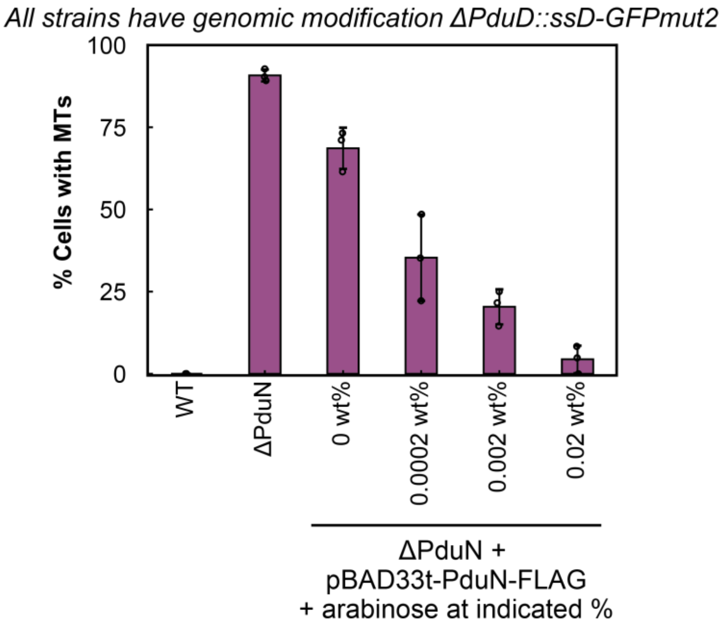

Supplementary Figure 3. Change in percentage of cells containing elongated, Pdu microtube (MT) structures as induction of PduN-FLAG off a plasmid is increased in a *pduN* knockout strain (ΔPduN). MT formation was visualized using a genomically integrated fluorescent reporter (ssD-GFP, integrated at the *pduD* locus [28]). WT indicates the wild type strain, and bars labeled with percentages are the PduN knockout strain with a plasmid encoding for FLAG-tagged PduN under an arabinose inducible promoter (pBAD33t-PduN-FLAG). Percentages indicate final arabinose concentration (w/w) in the culture. Center of error indicates the average over three biological replicates, and error bars indicate standard deviation over three biological replicates. Individual points represent percentage of cells with MTs within a single biological replicate. Source data are provided as a Source Data file.

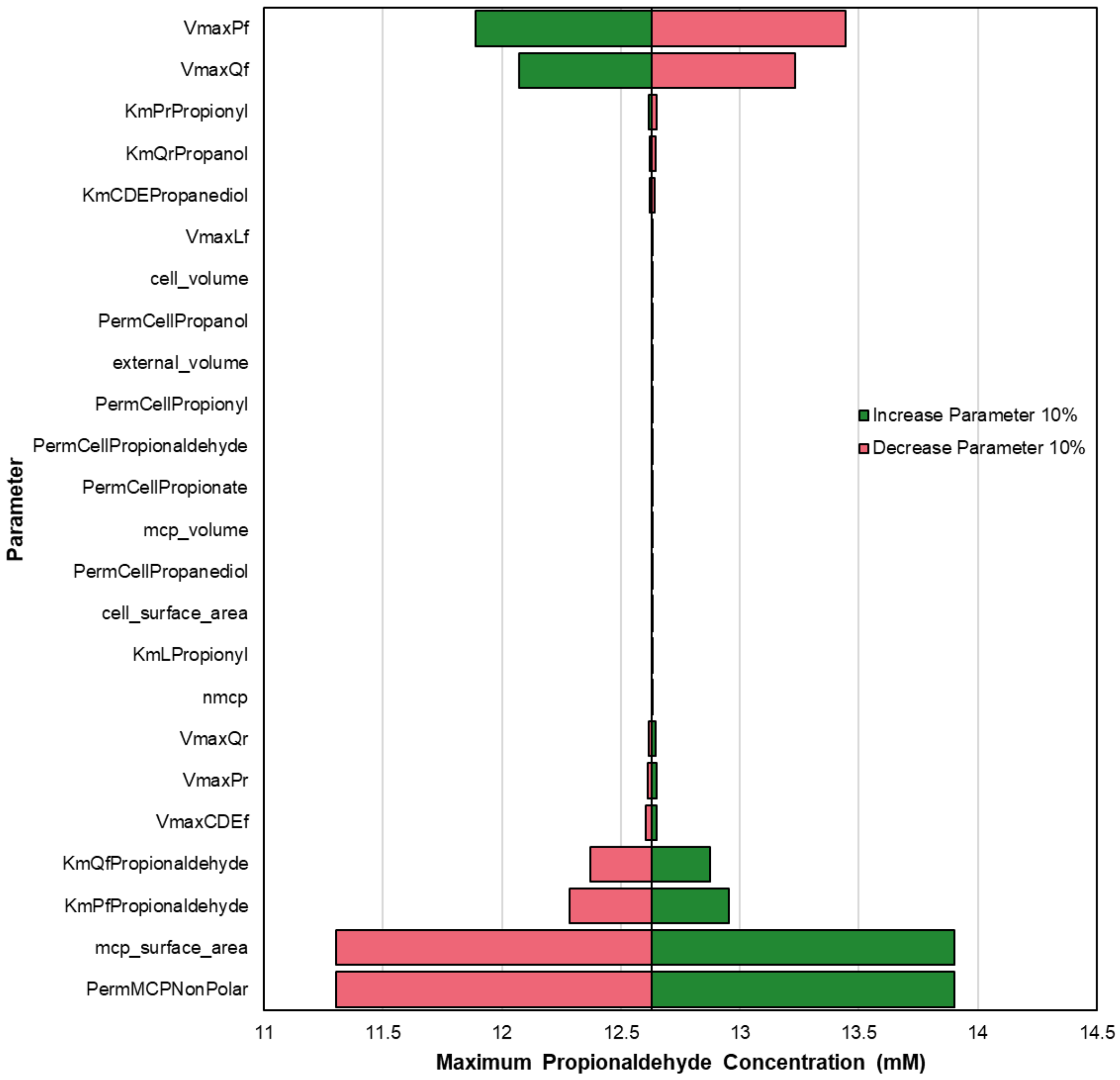

Supplementary Figure 4. Tornado plot showing changes in the maximum external propionaldehyde level in the MCP model with 10% increases and decreases in specified model parameters. Source data are provided as a Source Data file.

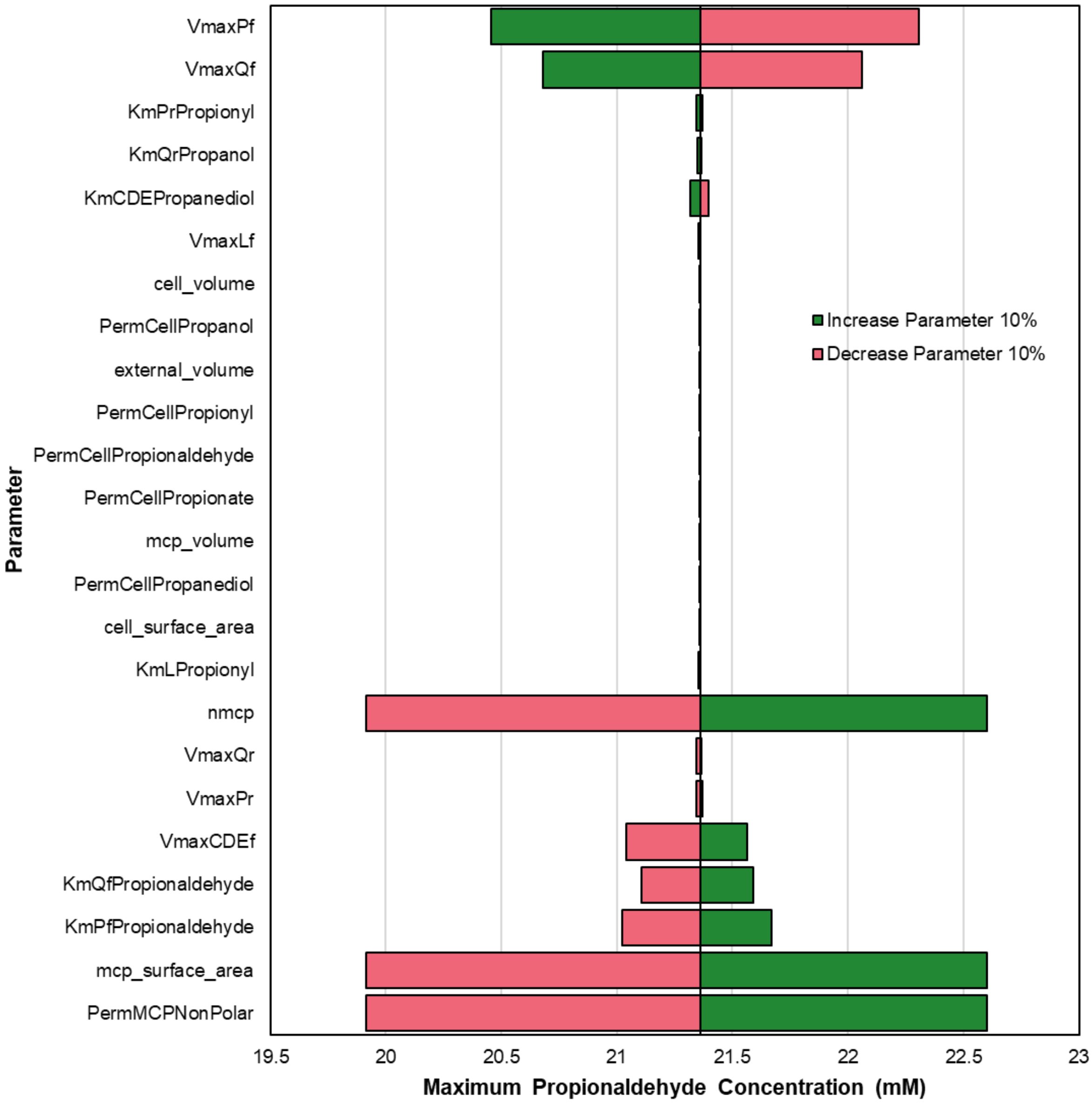

Supplementary Figure 5. Tornado plot showing changes in the maximum external propionaldehyde level in the microtube (MT) model (total MT volume equal to total MCP volume; enzyme concentration same as in MCP model; 190% increase in surface area compared to MCP surface area) with 10% increases and decreases in specified model parameters. Source data are provided as a Source Data file.

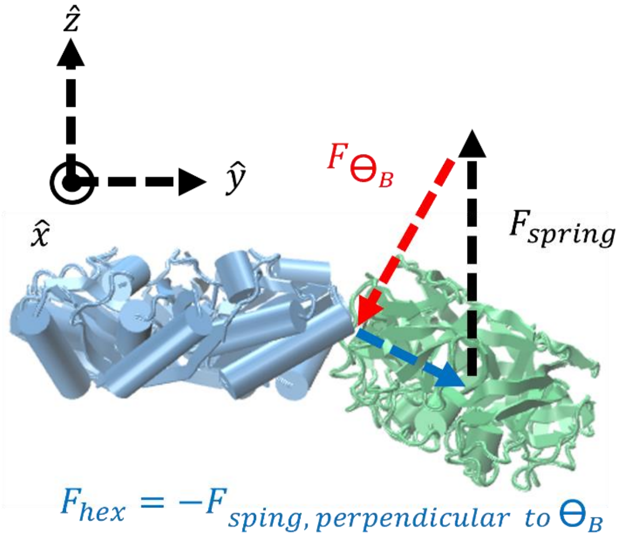

Supplementary Figure 6. Balance of forces in calculation of $F_{\theta_B}(z)$.

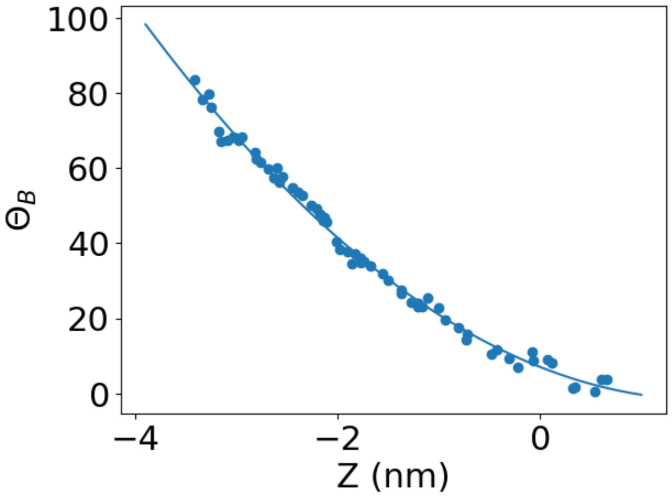

Supplementary Figure 7. Mapping between bending angle, θ_B (degrees), and the distance between the centers of mass of the pentamer and hexamer, z (nm). Source data are provided as a Source Data file.

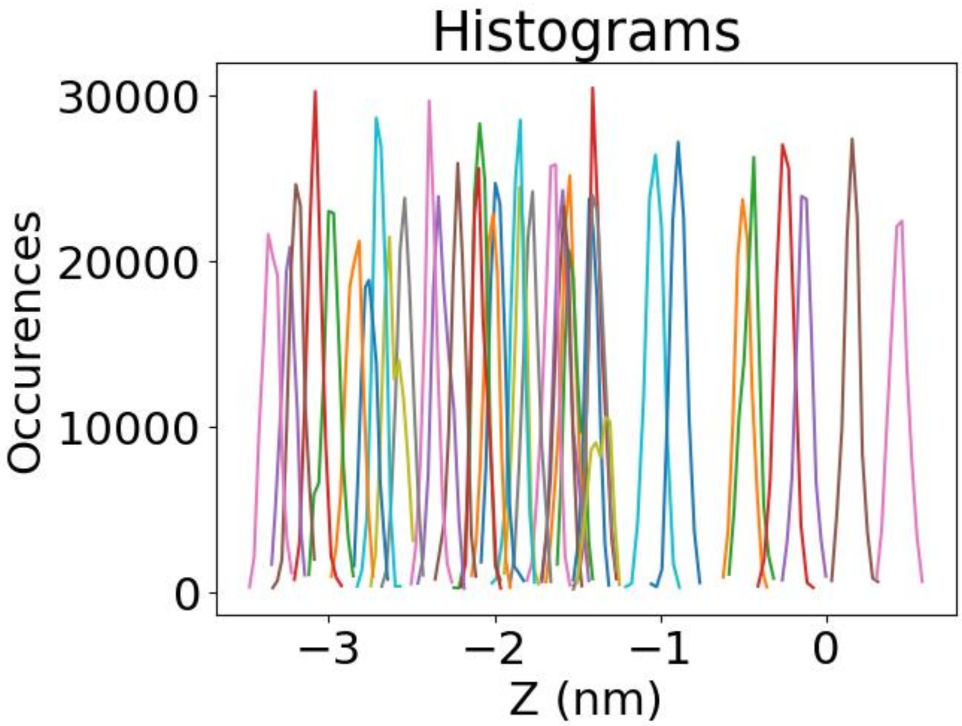

Supplementary Figure 8. Histograms showing the parallel “windows” used to calculate the potential of mean force. The overlap of the windows is shown by the distance between the centers of mass of the pentamer and hexamer, *z* (nm).

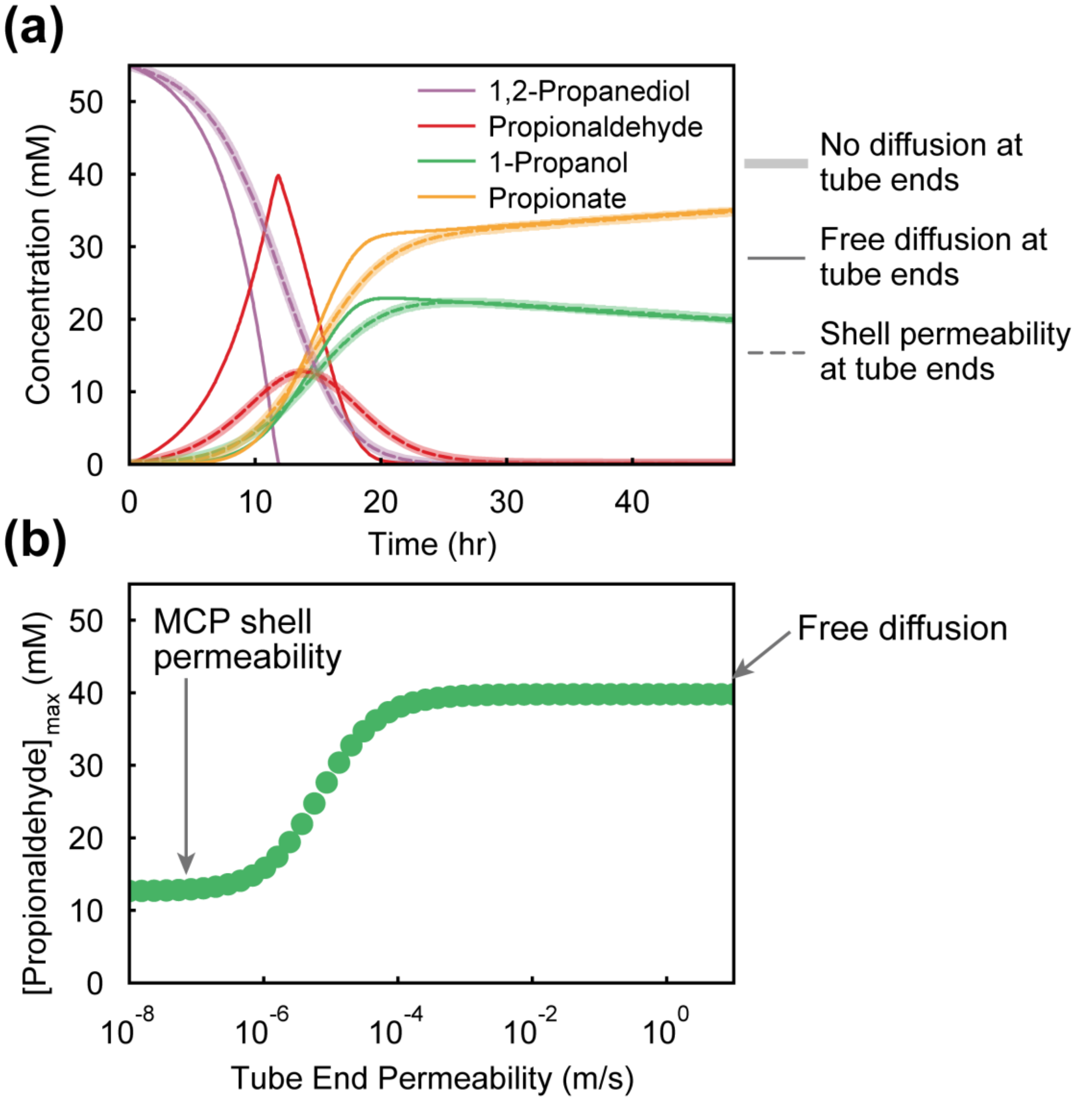

Supplementary Figure 9. Results of systems-level kinetic model on MTs that allow diffusion out of the ends, as described in Supplementary Method 2, Section 5, assuming 2.4 MTs per cell. (a) Plots of substrate concentrations in the external media for MTs with various permeability at the tube ends, compared to the case where no substrate diffusion is allowed at tube ends. Thick, slightly transparent lines are the base case, where there is no diffusion at tube ends, thinner and darker solid lines are free diffusion at tube ends and thinner and darker dashed lines represent the case where shell permeability is applied at tube ends. (b) Plot of maximum propionaldehyde concentration in the external media for MTs with different permeability at tube ends. Source data are provided as a Source Data file.

Supplementary references

[1] Páll, S., Abraham, M. J., Kutzner, C., Hess, B. & Lindahl. E., Tackling exascale software challenges in molecular dynamics simulations with GROMACS. in *Solving software challenges for exascale*. (ed. Markidis, S. & Laure, E.) 3–27 (Springer International Publishing Switzerland, London, 2015)

[2] Huang, J., *et al.* CHARMM36m: An Improved Force Field for Folded and Intrinsically Disordered Proteins. *Nat. Meth.* **14**, 71-73 (2017).

[3] MacKerell, Jr., A. D., *et al.* All-Atom Emprical Potential for Molecular Modeling and Dynamics Studies of Proteins. *J. Phys. Chem. B.* **102,** 3586-3616 (1998).

[4] Miyamoto, S. & Kollman, P. A. Settle: An Analytical Version of the SHAKE and RATTLE Algorithm for Rigid Water Models. *J. Comput. Chem.* **13,** 952-962 (1992).

[5] Darden, T., York, D. & Pedersen, L. Particle Mesh Ewald: An N·log(N) Method for Ewald Sums in Large Systems. *J. Chem. Phys.* **98**, 10089-10092 (1993).

[6] Essmann, U., Perera, L. & Berkowitz, M. L. A smooth particle mesh Ewald method. *J. Chem. Phys.* **103**, 8577-8593 (1995).

[7] Lee, J., *et al.* CHARMM-GUI Input Generator for NAMD, GROMACS, AMBER, OpenMM, and CHARMM/OpenMM Simulations Using the CHARMM36 Additive Force Field. *J. Chem. Theory Comput.* **12**, 405-413 (2016).

[8] Panganiban, B. *et al.* Random heteropolymers preserve protein function in foreign environments. *Science* **359**, 1239-1243 (2018).

[9] Qiao, B., Lopez, L. & Olvera de la Cruz, M. “Mirror”-Like Protein Dimers Stabilized by Local Heterogeneity at Protein Surfaces. *J. Phys. Chem. B* **123**, 3907-3915 (2019).

[10] Qiao, B., Jiménez-Ángeles, F., Nguyen, T. D. & Olvera de la Cruz, M. Water Follows Polar and Nonpolar Protein Surface Domains. *Proc. Natl. Acad. Sci. U. S. A.* **116**, 19274-19281 (2019).

[11] Qiao, B. & Olvera de la Cruz, M. Enhanced Binding of SARS-CoV-2 Spike Protein to Receptor by Distal Polybasic Cleavage Sites. *ACS Nano* **14**, 10616-10623 (2020).

[12] Jiang, T. *et al.* Single-chain heteropolymers transport protons selectively and rapidly. *Nature* **577**, 216-220 (2020).

[13] Jakobson, C. M., Tullman-Ercek, D., Slininger, M. F. & Mangan, N. M. A systems-level model reveals that 1,2-Propanediol utilization microcompartments enhance pathway flux through intermediate sequestration. *PLoS Comput. Biol.* **13**, e1005525 (2017).

[14] Sampson, E. M. & Bobik, T. A. Microcompartments for B12-Dependent 1,2-Propanediol Degradation Provide Protection from DNA and Cellular Damage by a Reactive Metabolic Intermediate. *J. Bacteriol*. **190**, 2966-2971 (2008).

[15] Bachovichin, W. W., Eagar, Jr., R. G., Moore, K. W. & Richards, J. H. Mechanism of action of adenosylcobalamin: glycerol and other substrate analogs as substrates and inactivators for propanediol dehydratase - kinetics, stereospecificity, and mechanism, *Biochemistry* **16**, 1082-1092 (1977).

[16] Leal, N. A., Havemann, G. D. & Bobik, T. A. PduP is a coenzyme-a-acylating propionaldehyde dehydrogenase associated with the polyhedral bodies involved in B12-dependent 1,2-propanediol degradation by Salmonella enterica serovar Typhimurium LT2, *Arch Microbiol.* **180**, 353-361 (2003).

[17] Cheng, S., Fan, C., Sinha, S. & Bobik, T. A. The PduQ Enzyme Is an Alcohol Dehydrogenase Used to Recycle NAD^+ Internally within the Pdu Microcompartment of *Salmonella enterica*. *PLoS ONE* **7**, e47144 (2012).

[18] Jakobson, C. M., Tullman-Ercek, D. & Mangan, N. M. Spatially organizing biochemistry: choosing a strategy to translate synthetic biology to the factory. *Scientific Reports* **8**, 8196 (2018).

[19] Sutter, M., Greber, B., Aussignargues, C. & Kerfeld, C. A. Assembly principles and structure of a 6.5-MDa bacterial microcompartment shell. *Science* **356**, 1293-1297 (2017).

[20] Greber, B. J., Sutter, M. & Kerfeld, C. A. The Plasticity of Molecular Interactions Governs Bacterial Microcompartment Shell Assembly. *Structure* **27**, 749-763 (2019).

[21] Klein, S. A., Majumdar, A. & Barrick, D. A Second Backbone: The Contribution of a Buried Asparagine Ladder to the Global and Local Stability of a Leucine-Rich Repeat Protein. *Biochemistry* **58**, 3480-3493 (2019).

[22] Yang, M. *et al.* Decoding the stoichiometric composition and organisation of bacterial metabolosomes. *Nat. Comm.* **11**, 1976 (2020).

[23] Able, K. R., Butler, M. H. & Wright, B. E. Cellular Concentrations of Enzymes and Their Substrates. *J. Theor. Biol.* **143**, 163-195 (1990).

[24] Kennedy, N. W. *et al.* Apparent size and morphology of bacterial microcompartments varies with technique. *PLoS ONE* **15**, e0226395 (2019).

[25] Orbach, E. & Finkelstein, A. The nonelectrolyte permeability of planar lipid bilayer membranes. *J. Gen. Physiol.* **75**, 427-436 (1980).

[26] Abraham, M. H., Whiting, G. S., Fuchs, R. & Chambers, E. J. Thermodynamics of solute transfer from water to hexadecane. *J. Chem. Soc., Perkin Trans.* **2**, 291-300 (1990).

[27] Schantz, M. M. & Martire, D. E. Determination of hydrocarbon-water partition coefficients from chromatographic data and based on solution thermodynamics and theory. *J. Chromatogr. A.* **391**, 35-51 (1987).

[28] Nichols, T. M., Kennedy, N. W. & Tullman-Ercek, D. A genomic integration platform for heterologous cargo encapsulation in 1,2-propanediol utilization bacterial microcompartments. *Biochem. Eng. J.* **156**, 107496 (2020).
