# Supplementary material for: Vertex protein PduN tunes encapsulated pathway performance by dictating bacterial metabolosome morphology
Source: Nat Commun. 2022 Jun 29;13:3746. doi: 10.1038/s41467-022-31279-3 (PMC9243111; doi:10.1038/s41467-022-31279-3)
Supplement: Supplementary file 3 — Description of Additional Supplementary Files [file 41467_2022_31279_MOESM3_ESM.pdf]

Description of Additional Supplementary Files

- Supplemental Data 1:
  List of all strains used in study

- Supplemental Data 2:
  List of all plasmids used in study

- Supplemental Data 3:
  List and sequences for all primers used in study
