# Supplementary material for: Vertex protein PduN tunes encapsulated pathway performance by dictating bacterial metabolosome morphology
Source: Nat Commun. 2022 Jun 29;13:3746. doi: 10.1038/s41467-022-31279-3 (PMC9243111; doi:10.1038/s41467-022-31279-3)
Supplement: Supplementary file 7 — Reporting Summary [file 41467_2022_31279_MOESM7_ESM.pdf]

nature portfolio

Corresponding author(s): Danielle Tullman-Ercek

Last updated by author(s): Jun 2, 2022

Reporting Summary

Nature Portfolio wishes to improve the reproducibility of the work that we publish. This form provides structure for consistency and transparency in reporting. For further information on Nature Portfolio policies, see our Editorial Policies and the Editorial Policy Checklist.

Statistics

For all statistical analyses, confirm that the following items are present in the figure legend, table legend, main text, or Methods section.

n/a	Confirmed	
☐	☒	The exact sample size (*n*) for each experimental group/condition, given as a discrete number and unit of measurement
☒	☐	A statement on whether measurements were taken from distinct samples or whether the same sample was measured repeatedly
☐	☒	The statistical test(s) used AND whether they are one- or two-sided<br>*Only common tests should be described solely by name; describe more complex techniques in the Methods section.*
☒	☐	A description of all covariates tested
☒	☐	A description of any assumptions or corrections, such as tests of normality and adjustment for multiple comparisons
☐	☒	A full description of the statistical parameters including central tendency (e.g. means) or other basic estimates (e.g. regression coefficient) AND variation (e.g. standard deviation) or associated estimates of uncertainty (e.g. confidence intervals)
☐	☒	For null hypothesis testing, the test statistic (e.g. *F*, *t*, *r*) with confidence intervals, effect sizes, degrees of freedom and *P* value noted<br>*Give P values as exact values whenever suitable.*
☒	☐	For Bayesian analysis, information on the choice of priors and Markov chain Monte Carlo settings
☒	☐	For hierarchical and complex designs, identification of the appropriate level for tests and full reporting of outcomes
☒	☐	Estimates of effect sizes (e.g. Cohen's *d*, Pearson's *r*), indicating how they were calculated

Our web collection on statistics for biologists contains articles on many of the points above.

Software and code

Policy information about availability of computer code

Data collection	Image Lab Sofware v 6.0.1 (Bio-Rad Laboratories), NIS-Elements BR 4.10.01 64-bit (Nikon), Gatan Digital Micrograph (Gatan, Inc.), UCSF Chimera version 1.13.1, GROMACS version 2016.3, BioTek Gen5 v 2.07, Agilent ChemLab in OpenLAB CDS ChemStation Edition for LC & LC/MS Systems Rev. C.01.07 SR2 (Agilent), Python 3.8.8 run with Spyder v 5.0.5 console, packages for Python: pandas (1.2.5), matplotlib (3.3.4), numpy (1.20.2), scipy (1.6.2), and csaps (1.0.4). Python code used for kinetic modeling provided on GitHub (https://github.com/cemills/MCP-vs-MT). This GitHub repository has been deposited in Zenodo.
Data analysis	Microsoft Excel 2016, ImageJ 1.53e, Visual Molecular Dynamics (VMD) for LINUXMD64 v 1.9.4a12 (December 21, 2017), Python 3.8.8 run with Spyder v 5.0.5 console, packages for Python: pandas (1.2.5), matplotlib (3.3.4), numpy (1.20.2), scipy (1.6.2), and csaps (1.0.4).

For manuscripts utilizing custom algorithms or software that are central to the research but not yet described in published literature, software must be made available to editors and reviewers. We strongly encourage code deposition in a community repository (e.g. GitHub). See the Nature Portfolio guidelines for submitting code & software for further information.

Data

Policy information about availability of data

All manuscripts must include a data availability statement. This statement should provide the following information, where applicable:

- Accession codes, unique identifiers, or web links for publicly available datasets
- A description of any restrictions on data availability
- For clinical datasets or third party data, please ensure that the statement adheres to our policy

Source data are provided with this paper. All strains used in this study are available upon request. We acknowledge use of PDBs 5V74 and 3NGK in this work.

Field-specific reporting

Please select the one below that is the best fit for your research. If you are not sure, read the appropriate sections before making your selection.

- [x] Life sciences
- [ ] Behavioural & social sciences
- [ ] Ecological, evolutionary & environmental sciences

For a reference copy of the document with all sections, see nature.com/documents/nr-reporting-summary-flat.pdf

Life sciences study design

All studies must disclose on these points even when the disclosure is negative.

Sample size	No sample size calculations were performed. As noted below, all experiments were performed in biological triplicate and all cultures used were seeded from a single, randomly selected colony, which provides some limited insight about the distribution of the final cell populations produced by these experiments. These procedures (performing experiments in biological triplicate and seeding cultures from a randomly selected colony) are standard in the field for experiments in which measurements are made on the population level. For cell measuring and counting of microscopy images, at least 100 cells were counted over three different biological replicates, as previously published work (Kennedy et. al J. Mol. Bio. 2021) showed that this number of observations was more than sufficient for producing a statistically significant difference in cell population measurements, as well as being easily realizable given the throughput of the assay.
Data exclusions	No data were excluded from analyses in this study.
Replication	All experiments were performed in biological triplicate (where each culture for a new experiment was inoculated from a separate single colony) to confirm reproducibility, except thin cell section TEM experiments. For thin cell section TEM experiments, we were limited by resources, and so multiple cells (at least 30) collected from a single biological replicate were observed and analyzed. Notably, these procedures for thin cell section TEM are standard in the field, especially when biological replicates of fluorescence microscopy corroborate all EM imaging results. All experiments presented were reproducible across different days.
Randomization	Colonies used to inoculate liquid cultures were selected at random for all experiments. Cells were sampled at random for measurement, as draws for analyses like microscopy were taken from well-mixed cultures.
Blinding	Blinding does not apply to our studies because none of our data was based on qualitative scoring metrics, nor does it involve animal or human subjects. Because all metrics described in this study were quantitative, they were not prone to biasing by individual investigators and thus blinding was deemed unnecessary.

Reporting for specific materials, systems and methods

We require information from authors about some types of materials, experimental systems and methods used in many studies. Here, indicate whether each material, system or method listed is relevant to your study. If you are not sure if a list item applies to your research, read the appropriate section before selecting a response.

Materials & experimental systems

n/a	Involved in the study	
	x	Antibodies
x		Eukaryotic cell lines
x		Palaeontology and archaeology
x		Animals and other organisms
x		Human research participants
x		Clinical data
x		Dual use research of concern

Methods

n/a	Involved in the study	
x		ChIP-seq
x		Flow cytometry
x		MRI-based neuroimaging

Antibodies

Antibodies used	PRIMARY: Monoclonal ANTI-FLAG® M2 antibody produced in mouse, clone M2, purified immunoglobulin (Purified IgG1 subclass), Sigma Aldrich (F3165), Lot Number SLCG330<br><br>SECONDARY: anti-Mouse IgG (H+L) Secondary Antibody, Horseradish Peroxidase conjugated produced in Goat, Invitrogen 32430, Lot Number VJ313743
Validation	PRIMARY: Per Certificate of Analysis, lot was confirmed: (1) to detect 2 ng of FLAG-BAP fusion protein by dot blot using chemiluminescent detection, (2) to produce two major bands with purity >90% when analyzed by microfluidic gel capillary electrophoresis, and (3) to detect a single band of protein on a western blot from an E. coli crude cell lysate.
